# Revision total en bloc spondylectomy for a recurrent aggressive vertebral haemangioma of the thoracic spine causing progressive thoracic myelopathy with segmental kyphosis

**DOI:** 10.1093/jscr/rjae461

**Published:** 2024-07-16

**Authors:** Igor Potparić, Peter Brumat, Klemen Bošnjak, Miha Vodičar

**Affiliations:** Faculty of Medicine, University of Ljubljana, Vrazov trg 2, 1000 Ljubljana, Slovenia; Department of Orthopaedic Surgery, Ljubljana University Medical Centre, Zaloška cesta 9, 1000 Ljubljana, Slovenia; Faculty of Medicine, University of Ljubljana, Vrazov trg 2, 1000 Ljubljana, Slovenia; Department of Orthopaedic Surgery, Ljubljana University Medical Centre, Zaloška cesta 9, 1000 Ljubljana, Slovenia; Department of Spine Surgery, Valdoltra Orthopaedic Hospital, Jadranska cesta 31, 6280 Ankaran, Slovenia; Faculty of Medicine, University of Ljubljana, Vrazov trg 2, 1000 Ljubljana, Slovenia; Department of Orthopaedic Surgery, Ljubljana University Medical Centre, Zaloška cesta 9, 1000 Ljubljana, Slovenia; Faculty of Medicine, University of Ljubljana, Vrazov trg 2, 1000 Ljubljana, Slovenia; Department of Orthopaedic Surgery, Ljubljana University Medical Centre, Zaloška cesta 9, 1000 Ljubljana, Slovenia

**Keywords:** aggressive vertebral haemangioma, recurrent vertebral haemangioma, total en bloc spondylectomy, thoracic myelopathy, kyphosis

## Abstract

Vertebral haemangiomas are common amongst primary benign tumours of the spine, usually asymptomatic and discovered incidentally, whereby symptomatic cases are rare. Aggressive vertebral haemangiomas, occurring even less frequently, are characterized by their expansion, resulting in pain and neural compression symptoms. Depending on presentation and severity, several treatment options exist, and when causing progressive neurological deficit, surgical decompression and resection is warranted. Despite local recurrence being rare, regular follow-ups to detect recurrence are advised. In case of recurrent aggressive vertebral haemangiomas, however, subsequent treatment strategy usually depends on a case-by-case consideration, whereby reports in the literature are lacking. We describe a case of a recurrent aggressive vertebral haemangiomas of the thoracic spine in a 20-year-old male causing progressive thoracic myelopathy with segmental kyphosis, treated with a revision total en bloc spondylectomy and a multilevel fixation with vertebral column reconstruction using radiolucent instrumentation.

## Introduction

Amongst primary benign tumours of the spine, vertebral haemangiomas (VH) are the most common, usually asymptomatic and discovered incidentally [1–3]. Approximately 10% of adults experience VH, with symptomatic cases occurring less frequently, while aggressive vertebral haemangiomas (AVH) represent only 0.9%–1.2% of all cases [1–3]. Although benign, aggressive local growth can cause pain and even neurological symptoms due to expansion, extraosseous extension toward the spinal canal, and compressive fractures, all potentially causing neural compression symptoms with myelopathy [2–5].

Depending on presentation and severity, several treatment options exist, including vertebroplasty, ethanol ablation, endovascular embolization, and, when causing progressive neurological deficit, surgical decompression with resection [1, 2, 4, 6, 7]. Despite local recurrence being rare, regular follow-ups to detect recurrence are advised [1, 6]. In case of recurrent AVH, however, subsequent treatment strategy usually depends on a case-by-case consideration, whereby reports in the literature are scarce [8].

## Case report

A 20-year-old male presented to our institution with clinical symptoms of myelopathy and a history of previous surgery for AVH at Th6. Upon admission, the patient reported a 1-month-long progressive paraesthesia in both thighs and back pain. Neurological examination revealed a right-sided extension plantar reflex and residual hypoesthesia in the right anterior thigh, with no significant motor impairment observed (ASIA score D, VAS pain score 7).

**Figure 1:**
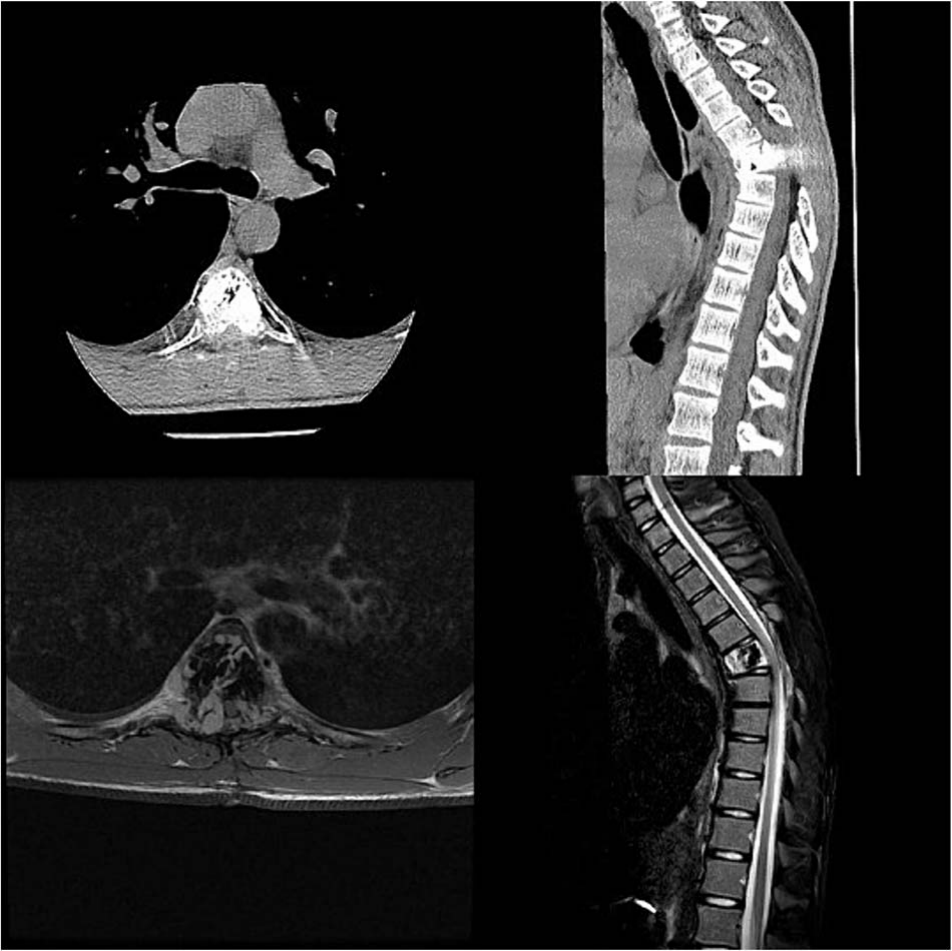
Follow-up CT imaging revealed recurrent AVH of the Th6 vertebrae, MRI scan showing AVH expanding into the spinal canal and causing myelopathy.

**Figure 2:**
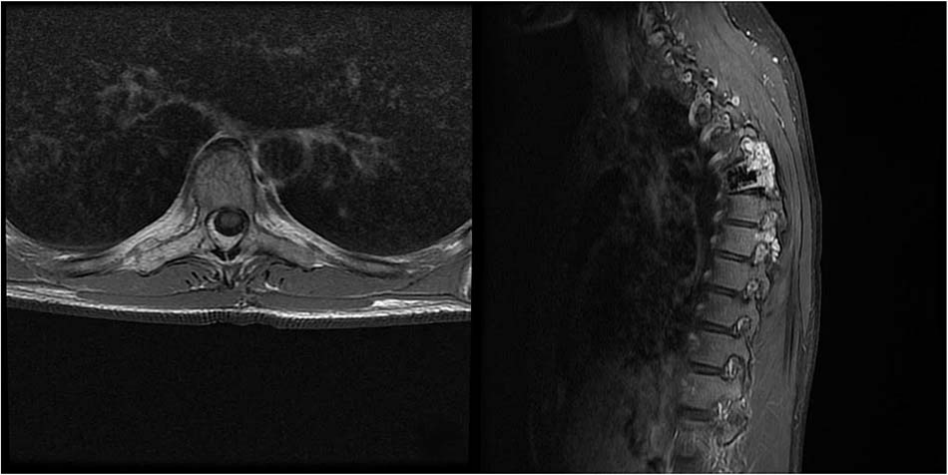
MRI scan of the thoracic spine showing a concomitant haemangioma in the right transverse and articular process of Th8.

Three and a half years prior to admission to our institution, the patient underwent emergent Th6 vertebroplasty with laminectomy for a radiologically characteristic and histologically confirmed Th6 AVH causing acute thoracic myelopathy with a week-long paresis of the right foot dorsiflexion, performed by another surgeon. A progressive kyphotic deformity of the Th6 vertebrae was observed on radiographic follow-ups, whereby the hypoesthesia on the right anterior thigh persisted (ASIA score D, VAS pain score 7). Follow-up MRI and CT imaging revealed recurrent AVH of the Th6 vertebrae expanding into the spinal canal, leading to newly recognized worsening of myelopathy (Fig. 1). Additionally, a concomitant haemangioma was observed in the right transverse and articular process of Th8 (Fig. 2).

**Figure 3:**
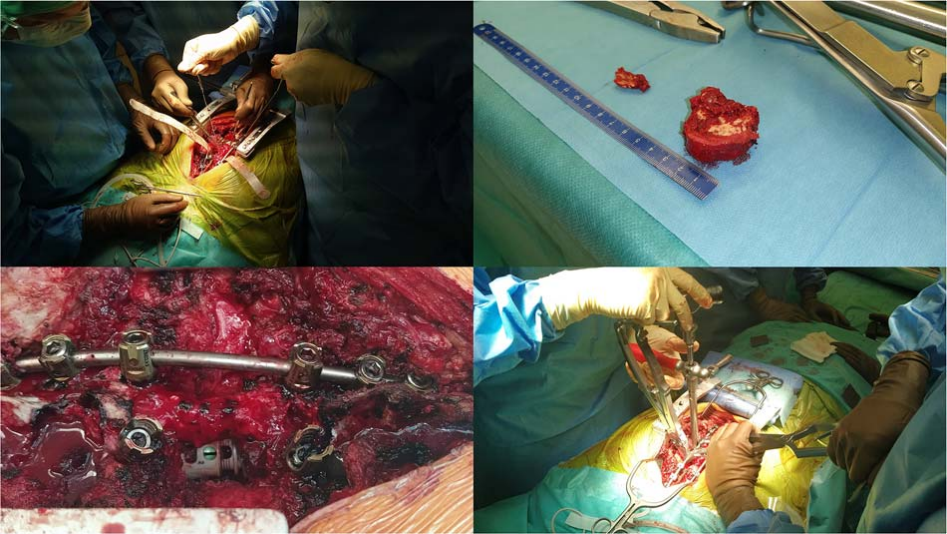
Intraoperative pictures of Th6 removal, instrumentation, and cage application.

**Figure 4:**
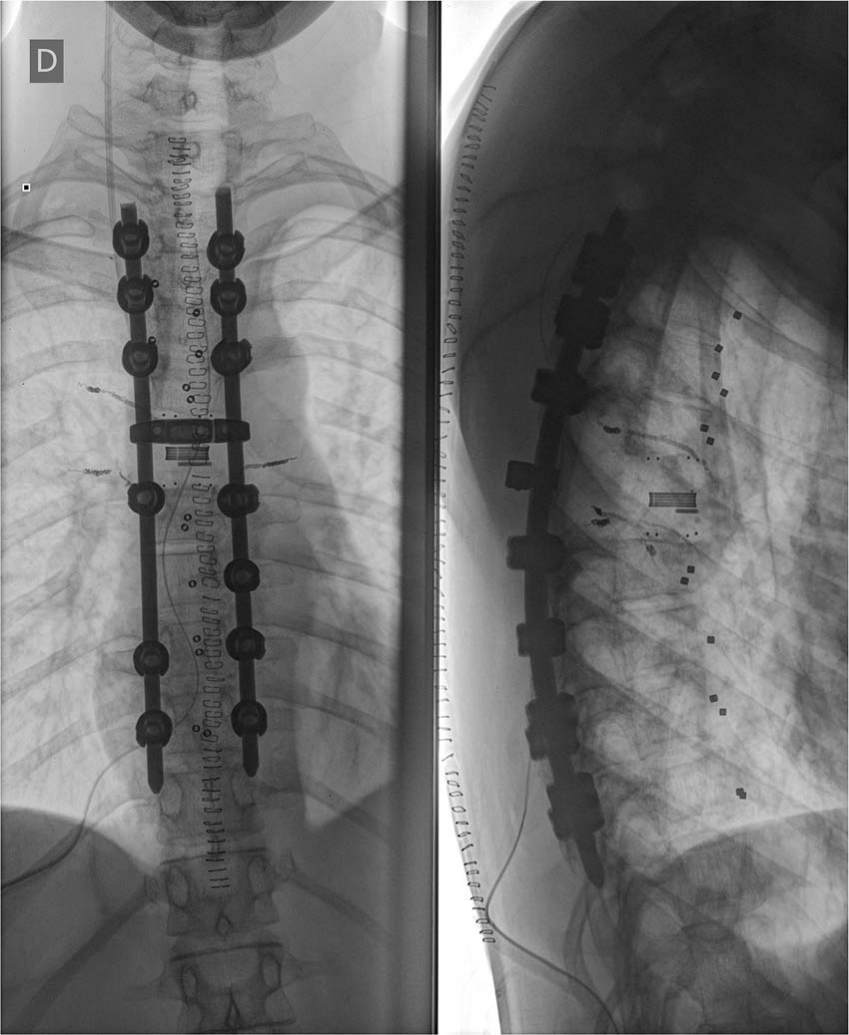
Postoperative X-ray imaging immediately after surgery; anteroposterior view (left) and lateral view (right).

**Figure 5:**
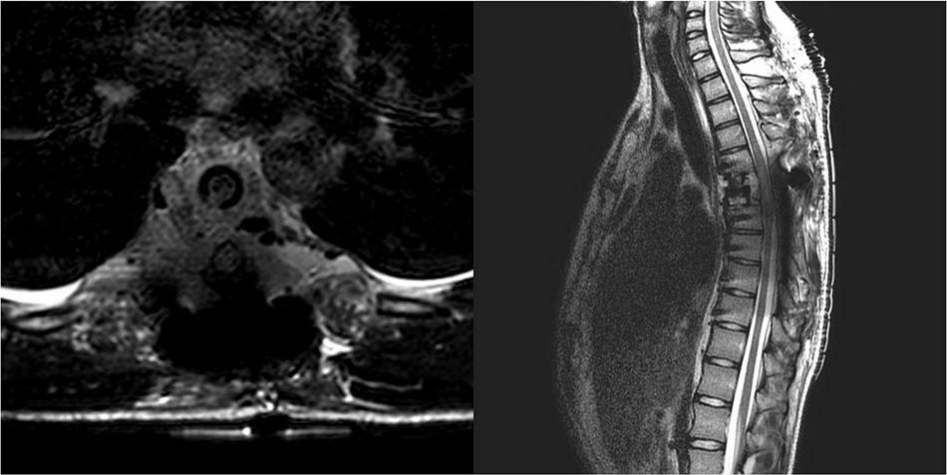
Immediate postoperative MRI showing no residual signs of thoracic spinal cord compression.

**Figure 6:**
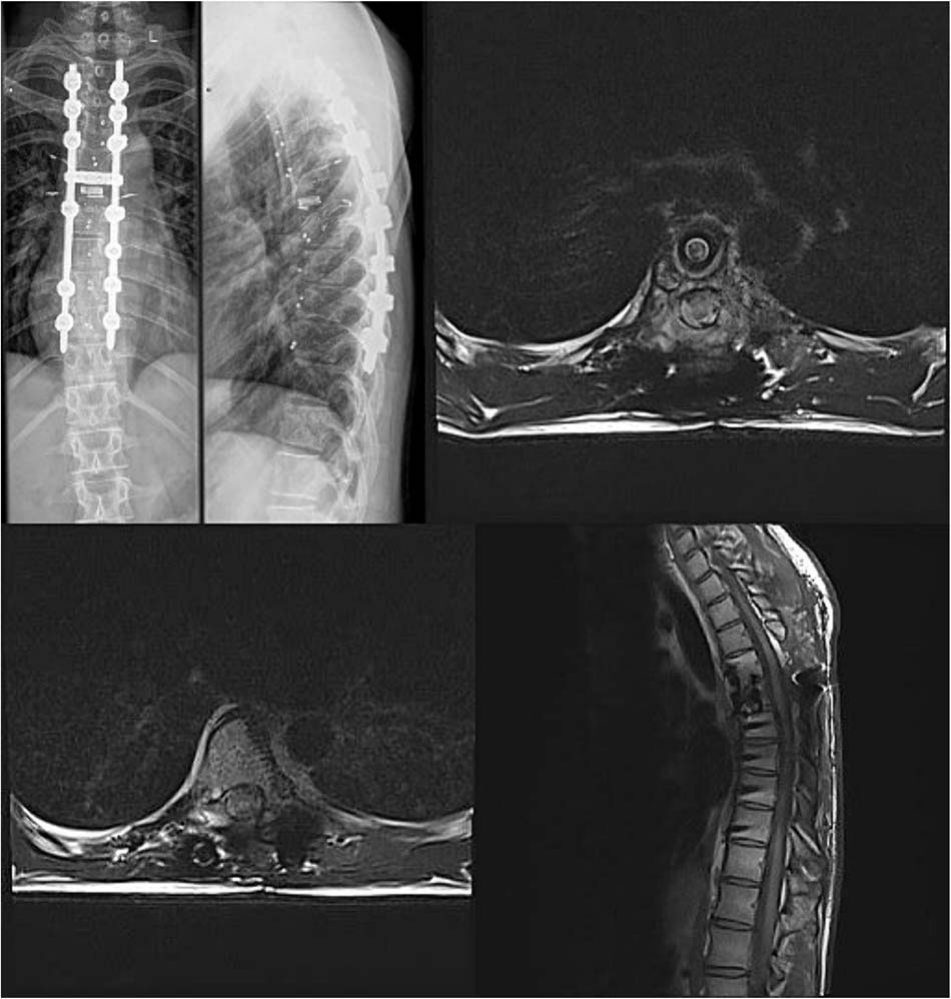
Final follow-up X-Ray scan and MRI imaging showing no radiological signs of spinal instability or myelopathy.

We decided to perform a revision total en bloc spondylectomy at Th6, along with resection of the Th8 pedicle and costovertebral joint, with a partial resection of the adjacent rib. Posterior spinal fixation from Th3 to Th10 was performed using radiolucent carbon screws, and the Th6 body was reconstructed using a radiolucent expandable cage (Figs 3 and 4). During the surgery, MEPs and SEPs were lost, with the patient waking up paraplegic (ASIA score B, VAS pain score 5). Immediate postoperative MRI showed no residual signs of thoracic spinal cord compression due to tumour or haemathoma (Fig. 5). The patient’s neurological status substantially improved the day after surgery, and thereafter, gradually improving over the next few days, allowing the patient to walk independently within 10 days (ASIA score D, VAS pain score 3). A multidisciplinary council decided against postoperative radiotherapy to avoid aggravating neurological symptoms by possibly causing additional damage to the myelopathic spinal cord. On the 11th postoperative day, the patient reported dyspnoea, and urgent CT angiography revealed peripheral pulmonary embolism, which was managed utilizing Deltaparine. Subsequent postoperative course was uneventful, leading to his discharge on the 15th postoperative day, ambulating independently using a walker. MRI follow-up was conducted 6 months postsurgery, followed by yearly checks. At the last follow-up, 2.5 years after the second surgery, there were no signs of tumour recurrence, and the patient exhibited no gait disturbance, back pain, or radiological signs of spinal instability (Fig. 6) (ASIA score E, VAS pain score 0).

## Discussion

VHs are prevalent among adults, yet only about 1% exhibit expansion (known as AVH) and symptoms such as pain, pathologic fractures, and neural compression. [1, 3]. The thoracic spine is the predilected region, a characteristic shared with our case [4, 9]. In contrast, our patient’s primary symptoms and the index operation occurred during childhood, a rarity in this context [4, 5].

Treatment options for AVH vary based on presentation and severity, with surgical decompression, with or without instrumentation, being the preferred approach in cases of neural compression [1, 4, 5, 10]. There are some recommendations on the management of VHs [7, 8, 11]. Despite most treatment options providing good outcomes, recurrence is still an issue with varying rates, depending on the treatment method [1]. The surgical strategy for AVHs remains controversial due to its rarity, with decision-making strategy for the management and the choice of surgical intervention varying dependently over the location and the extension of the tumour [6, 8, 12]. Moreover, reports on treatment strategies for recurrent AVH cases are lacking. According to Goldstein *et al.* [1], recurrence rates vary and cannot be predicted based on the procedure, but can be minimized by using diagnostic and classification criteria, which could help plan procedures and progress tracking.

Most authors agree that, when possible, preoperative arterial embolization should be performed to minimize blood loss during surgery [1, 2, 4, 5, 10, 13, 14], yet as a stand-alone procedure, it may only be moderately effective as a definitive treatment due to tumour recanalization [1]. The extent of resection when dealing with recurrence in revision surgery setting is questionable, whereby most authors favour piecemeal gross-resection and en bloc resection as definitive treatment options with varying amount of blood loss [1, 2, 4, 5, 10]. A modern, real-time monitoring navigation-guided methods show promise in enabling better results in reducing residual AVH while maintaining a safer and easier tumour resection [15].

Showing excellent pain control in various studies, with minimal toxicities, radiation therapy may be an acceptable treatment option in aggressive or inoperable cases and is also recommended in cases following partial resection and extensive lesions, bearing a recurrence rate as high as 30%–50% without the addition of radiotherapy after subtotal resection [12]. A combination of vertebroplasty and radiation therapy may also present a treatment alternative.

To conclude, cases of recurrent AVH are rare and subsequent treatment depends on a case-by-case consideration. In the presented case report, en bloc resection of the vertebral body was chosen to minimize the chances of renewed recurrence. Our decision has thus far demonstrated considerable clinical success; however, the adequacy of this treatment will ultimately be determined over time.

## Conflict of interest statement

All authors declare no conflict of interest.

## Funding

This research did not receive any specific grant from funding agencies in the public, commercial, or not-for-profit sectors.

## Informed consent statement

The patient provided written informed consent for his participation in the study and for his anonymized data to be published in this article.

## Data availability

This manuscript presents a case report, and therefore, the data are not available publicly or upon request to protect the privacy and identity of the patient.
